# Prevalence of vaccine and non-vaccine human papillomavirus types among women in Accra and Kumasi, Ghana: a cross-sectional study

**DOI:** 10.1186/s12905-021-01511-1

**Published:** 2021-10-26

**Authors:** Oksana Debrah, Francis Agyemang-Yeboah, Emmanuel Timmy Donkoh, Richard Harry Asmah

**Affiliations:** 1 Institutional Care Division, Ghana Health Service Headquarters, Stadium Post Office, Post Office Box SD 329, Accra, Ghana; 2 Department of Molecular Medicine, Kwame Nkrumah University of Science and Technology, Kumasi, Ghana; 3 Department of Basic and Applied Biology, University of Energy and Natural Resources, Sunyani, Ghana; 4 University of Health and Allied Sciences, Ho, Ghana; 5 Department of Science Laboratory Technology, Accra Technical University, Accra, Ghana

**Keywords:** Human papillomavirus, Genotype distribution, HPV vaccine, Ghana

## Abstract

**Background:**

Human Papillomavirus (HPV) infection is the main etiological factor for pre-invasive and invasive cervical cancer. HPV type-specific vaccination is being widely recommended to control the burden of disease, but the genotype-specific distribution of HPV may vary in different countries. The aim of the study was to determine the prevalence and distribution of HPV genotypes among women attending reproductive health services in Ghana, their associated risk factors, and to assess the potential coverage of identified HPV genotypes by three licensed vaccines among these women.

**Method:**

Women presenting for reproductive health services in two regional hospitals in Accra and Kumasi from October 2014 to March 2015 were conveniently recruited into the study (n = 317). HPV-DNA detection and genotype identification were carried out by a nested multiplex PCR assay that combines degenerate E6/E7 consensus primers and type-specific primers for the detection and typing of eighteen HPV genotypes. Cytology was performed to screen women for cervical cancer lesions. Risk factors for HPV infection were analyzed by logistic regression. Statistical significance was accepted for *p* < 0.05.

**Results:**

The age of study participants ranged from 21 to 76 years. Among women positive for HPV, 35.0% were infected with high-risk HPV, 14.5% with probable high-risk HPV, and 17.0% with low-risk HPV. The prevalence of HPV 16/18 was 8.2%, HPV 6/11/16/18 was 9.1% and HPV 6/11/16/18/31/33/45/52/58 was 28.4%. The most prevalent among HR-HPV were types 52 (18.3%) and 58 (8.8%). HPV positivity may be associated with educational background (*p* < 0.001), age at first pregnancy (*p* = 0.028), and age at coitarche (*p* = 0.016).

**Conclusions:**

Our study revealed a high prevalence of HR-HPV infection among women. The high prevalence of HR HPV indicates that multivalent vaccines will be useful for controlling HPV burden in general population contexts. The distribution of HPVs in this population suggests that of the three currently available vaccines the nonavalent vaccine, which protects against seven HPV types in addition to HPV 16 and 18, has the highest coverage of HPV infections among Ghanaian women. Healthcare officials planning to reduce the transmission of HPV and cervical cancer must consider the coverage of the nonavalent vaccine as an advantage.

**Supplementary Information:**

The online version contains supplementary material available at 10.1186/s12905-021-01511-1.

## Introduction

Human papillomavirus (HPV) infection is the most common sexually transmitted viral infection worldwide [1]. Over 200 HPV types have been identified and have been completely sequenced [2]. Genital HPV types are classified as high risk (HR), probable high risk (PHR) or low risk (LR) according to the degree or likelihood associated with developing cervical cancer [1, 2]. Approximately 50 genotypes are known to be oncogenic or HR and are correlated with invasive cervical cancer [3]. Among the 15 most common types known to be carcinogenic, HPV16 and HPV18 are responsible for approximately 70% of cervical cancer worldwide [4, 5]. Other oncogenic HPV types, including HPV 31, 33, 45, 52, and 58, are estimated to be responsible for another 18% of cervical cancers [6].

In Ghana, cervical cancer is ranked as the second most common cancer with an estimated incidence of 27.4 per 100,000 women [7, 8]. Around 2797 women are diagnosed yearly and approximately 1699 deaths occur from cervical cancer [7, 8]. The World Health Organization (WHO) predicts that there will be 5000 new cases of cervical cancer and 3361 cervical cancer deaths will occur annually in Ghana by 2025 [9].

HPV vaccination for young girls and cervical screening programs for older women can be an effective strategy to prevent cervical cancer [10, 11]. Cervical screening programs, such as cervical cytology and visual inspection with acetic acid (VIA), are available but not mandatory in Ghana. The cervical screening programs are only effective on cervical cancer mortality, if a high proportion of women participate [12]. Moreover, it has been difficult to implement screening programs for cervical cancer in Ghana as well as in most sub-Saharan African countries, partly due to competing health needs such as HIV, malaria, tuberculosis and malnutrition [13, 14].

Currently, three HPV-vaccines have been approved: quadrivalent Gardasil®, Cervarix®, and a nonavelent Gardasil® consisting of type-specific HPV L1 virus-like particles (VLPs) that induce type-restricted protection [15–17]. All three vaccines prevent HPV-16 and HPV-18 infection. Quadrivalent-Gardasil® also protects against HPV-6 and HPV-11 whereas the nonavalent Gardasil® targets an additional five HPV types (HPV-31, 33, 45, 52, and 58) [18]. These vaccines may also have some cross-protection against other less common HR-HPV types [19, 20]. HPV-vaccination is documented to be safe, immunogenic, and associated with decreased HPV infection rates and lowered risk of HPV related diseases [21–23].

In Ghana, the quadrivalent-Gardasil® vaccine was piloted into the school-based vaccination program for 6000 girls aged years, in four districts in the Northern and Greater Accra Regions with support from the GAVI Alliance in November 2013 [24]. Meanwhile, the vaccine is available to the public for vaccination at a cost of approximately USD 50 per dose, potentially rising to USD 150 for three doses [25]. In spite of their availability for almost a decade, the uptake of HPV vaccines in Ghana has been poor. A number of reasons such as exclusion from services covered by universal health insurance and the low patronage of reproductive health services by adolescent girls may be cited [25–27]. However, the cost of HPV vaccines is a well-known barrier to vaccine accessibility in Sub-Saharan Africa and more innovative pricing solutions are constantly sought [25, 28, 29]. The large-scale deployment of vaccines with a broad coverage of common HPV types in a single-dose regimen could represent a sustainable, cost-effective, preventive strategy and potentially increase uptake of vaccination [30]. However, very few studies on HPV types not targeted by the present HPV-vaccines among Ghanaian women are available. The aim of our study was to determine the prevalence and distribution of HPV genotypes among Ghanaian women, their associated risk factors and to assess the potential coverage of identified HPV genotypes by available licensed vaccines.

## Materials and methods

### Study design, population and sampling technique

Between October, 2014 and March, 2015, women presenting to the Cervicare/Reproductive Health Clinics at the Kumasi South Regional Hospital in Kumasi, Ashanti Region and Greater Accra Regional Hospital in Accra, Greater Accra Region, Ghana, for reproductive counselling, routine family planning, visual screening, Pap smear testing and other support services were invited to participate in this hospital-based study. The minimum sample size was determined using the Cochran formula [31] for estimating single population proportions. In a previous study, the prevalence of HPV 16/18 was 5% [32] and the utilization of reproductive health services was approximately 8% for women of fertility age (WIFA) in study areas [33]. Based on a confidence level of 95% (z = 1.96), 5% margin of error (d), and 10% non-response rate, a minimum sample size of 317 was required. The convenient sampling technique was employed to enroll participants. Criteria for exclusion included: pregnancy; < 20 years-old; actively menstruating on the day of sample collection; history of hysterectomy or conization; history of Pap smear and prior HPV vaccination. Participants were fully informed about the purpose, procedures, risks, and benefits of participating in this study and informed consent was obtained from all subjects.

The study was approved by the Committee on Human Research Publication and Ethics (CHRPE), Kwame Nkrumah University of Science and Technology, School of Medical Sciences (KNUST-SMS) and Komfo Anokye Teaching Hospital (KATH) (CHRPE/AP/115/14), Kumasi, Ghana and Ghana Health Service Ethical Review Committee, Research and Development Division (GHS-ERC: 07/03/2014) in accordance with the revised Helsinki Declaration of 1964 (revised 2000) on ethical principles for medical research involving human subjects.

### Data collection

At enrolment, participants completed a questionnaire and provided data on sexual behavior, reproductive history, contraceptive practice, smoking habits, history of sexually transmitted disease, screening history, and various measures of demographics and socioeconomic status (e.g., occupation, education), cervical cancer screening options and HPV vaccination.

### Sample collection and processing

Two cervical samples (one each for Pap and HPV testing) were collected by trained nurses. Cervical specimens for HPV DNA test were suspended in a proprietary DNA solution (Biomatrica Co., San Diego, USA) for DNA preservation at room temperature until DNA extraction.

For cytological examination, all samples were stained using the modified Papanicolaou technique [34]. Results from cervical cytology specimens were reported according to the 2014 Bethesda Classification System for reporting cervical cytology [35].

### Genomic DNA extraction

Genomic DNA from cervical swabs was extracted using commercial spin-column based QIAamp Mini kit (QIAGEN, Hilden, Germany), according to the manufacturer’s instructions. The DNA was aliquoted in duplicate (25 µl each into separate 2 ml Eppendorf tubes) and stored at − 20 °C until further analysis. The concentration of extracted DNA was determined by spectrophotometry at 260 nm (Nano-Drop 2000c spectrophotometer, Thermo Fisher Scientific, USA). All samples were pre-screened with the human β-globin primers PCO3+/PCO4+ to assess sample integrity [36]. Briefly, for a PCR volume of 25 ml, 1–4 ml of the DNA lysate and 25 pmol of each human beta-globulin consensus primers PCO3+ and PCO4+ (Integrated DNA Technologies, Inc, USA) were used. Purified DNA was used in PCR as templates to amplify target regions.

### DNA HPV analysis

HPV-DNA detection and identification of the genotypes was carried out by nested multiplex PCR (NMPCR) as described by Sotlar et al. [37]. Briefly, a single consensus forward primer (GP-E6-3F) and two consensus back primers (GP-E7-5B and GP-E7-6B) were used for the general primer PCR. The PCR reaction mix of 50 µl contained 10X PCR buffer, 2.5 mM MgCl_2_ 200 µM of each of the four deoxyribonucleoside triphosphates (dNTP), 15 pmols of each E6/E7 consensus primers and 1.25 units of Taq polymerase enzyme (New England Biolabs Inc., UK). Four microlitres (4 µl) of DNA extracts was used as template for the amplification reactions. This was carried out using a thermal cycler (Stratagene Robocycler Gradient 96, Roche Molecular System Inc, USA). The cycling parameters for the first round PCR with E63F/E75B/E76B consensus primers were as follows: 94 °C for 4 min (initial denaturation), followed by 40 cycles of 94 °C for 1 min (denaturation), 40 °C for 2 min (annealing), 72 °C for 2 min (extension) and a single final elongation step of 72 °C for 10 min. In the second round PCR, 2 µl of first round PCR product, 15 pmols of forward and reverse primers for genotyping were used. Primers for the identification of high-risk genotypes 16, 18, 31, 33, 35, 39, 45, 51, 52, 56, 58, 59; probable high-risk genotypes 66 and 68, and low-risk genotypes 6/11, 42, 43, and 44 were used in four cocktails, each containing four to five different primer pairs. The other parameters that were used in the first round PCR mix were maintained. However, the cycling parameters were as follows: 94 °C for 4 min followed by 35 cycles of 94 °C for 30 s, 56 °C for 30 s, 72 °C for 45 s and a single final elongation step of 72 °C for four minutes.

### Detection of PCR products

The amplified products were detected by agarose gel electrophoresis (2%), containing 0.5 µg/ml EZ-Vision® Bluelight DNA dye (AMRESCO, LLC USA). Ten microlitres of each sample was added to 2 µl of orange G (5X) gel loading dye for the electrophoresis. Hundred base pair DNA molecular weight marker (New England Biolabs Inc., UK) was run alongside the PCR products. The gel was prepared and electrophoresed in 1X TAE buffer using a mini gel system at 100 V for one hour. The gels were viewed in a benchtop UV illuminator (UVP, LLC, Upland, CA, USA) and photographed using Canon camera Sx230 HS.

All methods were carried out in accordance with relevant guidelines and regulations.

### Statistical analysis

Data obtained from the questionnaire was checked for accuracy, stored in Microsoft Excel 2010 software (Microsoft Corporation, Redmond Campus, Washington DC, USA) and analyzed using the Statistical Package for Social Scientists (SPSS) version 22. Qualitative variables were described by simple counts and percentages. The confidential interval (95%CI) for the prevalence was determined using the Cochran formula [31]. Quantitative variables were represented as mean ± SD. Any HPV infection/overall HPV infection was defined as any or inclusive of all 18 HPV types. HPV genotypes were grouped according to the licensed vaccines: HPV-16/18, HPV-6/11/16/18 and HPV-6/11/16/18/31/33/45/52/58. Type-specific reporting on HPV genotypes accounted for each infection independently in women with multiple infections. The distribution of HPV genotypes was summarized using frequency distributions. The relation of HPV genotypes with demographic, gynecological and behavioral variables were examined by logistic regression. A *p* value < 0.05 was considered statistically significant.

## Results

### Study population characteristics

In total, complete the consent process and questionnaire. 317 women were screened for HPV genotypes after documenting informed consent and questionnaire administration. Analysis of the study population (n = 317) by sociodemographic characteristics revealed that more than half (67.8%: 215/317) of the respondents were between ages 25 and 44 years, married or cohabiting (64.7%: 205/317) (Table 1). Most were economically active (86.8: 275/317) with an educational level described as senior high school and higher (59.3%: 188/317). Concerning their sexual and reproductive characteristics, most (76.9%: 244/317) of the respondents were multiparous women who had their first pregnancy after age of 18 years (73.2%: 232/317) and had their first sexual contact before age 20 years (60.9%: 193/317).

**Table 1:** Demographic characteristics of study population

Characteristics	Number	Percentage (%)
*Age group (years)*		
**≤ **25	8	2.5
25–44	215	67.8
45–64	87	27.4
≥ 65	7	2.2
*Education*		
Below SHS	188	59.3
SHS and above	129	40.7
*Marital status*		
Married/cohabiting	205	64.7
Single/widowed/divorced	112	35.3
*Occupation*		
Economically active^a^	275	86.8
Not economically active^b^	42	13.2
*Age at first pregnancy (years)*		
≤ 17	27	8.5
18–21	97	30.6
22–25	76	24.0
> 25	59	18.6
Never pregnant	37	11.7
Do not remember	21	6.6
*Gravidae*		
0	37	11.7
1	36	11.4
≥ 2	244	76.9
*Parity*		
0	63	19.8
1	52	16.4
2–4	162	51.1
> 5	40	12.6
*Age of coitarche (years)*		
≤ 15	22	6.9
16–20	171	53.9
21–25	42	13.2
≥ 26	20	6.3
Do not remember	62	19.6
*Number of life time sex partners*		
1	112	35.3
2+	205	64.7
*Alcohol consumption*		
Yes	135	42.6
No	182	57.4
*Condom use*		
Yes	108	34.1
No	209	65.9

*SHS* Senior high school

^a^Economically active—have any job

^b^Not economically active—have no job, pensioner or housewife

### Overall HPV prevalence and type distribution

The estimated burden of non-specific HPV infections among women was 43.5% (95% CI 37.5–48.6%) (Table 2). The prevalence of high-risk (HR) HPV genotypes (types 16, 18, 31, 33, 35, 39, 45, 51, 52, 56, 58 and 59) was 35.0% (95% CI 29.0–40.4%), low risk-(LR) HPV genotypes (types 6/11, 42, 43 and 44) was 17.0% (95% CI 12.9–21.2%), while that of probable high-risk (PHR) HPV genotypes (types 66 and 68) was 14.5% (95% CI 10.7–18.3%). The HR HPV group caused the majority of the HPV infections (80.4% of HPV positive cases), followed by the LR HPV group (39.1% of HPV positive cases) and PHR HPV group (29.7% of HPV positive cases).

**Table 2:** Prevalence of human papilloma virus (HPV) and vaccine covered genotypes among women

Parameter	Frequency*	Proportion	Proportion	Proportion	SE	95% CI	
		(%)^a^	(%)^b^	(%)^c^		Lower	Upper
HPV positive	138	43.5			2.9	37.9	48.6
*Type of infection*							
Single	59	18.6	42.8		2.1	14.2	23.0
Multiple	79	24.9	57.2		2.5	20.2	30.6
*Risk category*							
High risk	111	35.0	80.4		2.8	29.6	40.4
Probable high risk	46	14.5	33.3		2.0	10.7	18.3
Low risk	54	17.0	39.1		2.2	12.9	21.2
*High-risk HPV genotype*							
HPV-16	14	4.4	10.1	12.6	1.2	2.2	6.6
HPV-18	13	4.1	9.4	11.7	1.1	2.2	6.3
HPV-31	12	3.8	8.7	10.8	1.1	1.9	6.3
HPV-33	2	0.6	1.4	1.8	0.5	0.0	1.6
HPV-35	17	5.4	12.3	15.3	1.3	2.8	7.9
HPV-39	10	3.2	7.2	9.0	1.0	1.6	5.4
HPV-45	13	4.1	9.4	11.7	1.1	2.2	6.3
HPV-51	7	2.2	5.1	6.3	0.8	0.6	3.8
HPV-52	58	18.3	42.0	52.3	2.1	14.2	22.4
HPV-56	8	2.5	5.8	7.2	0.9	0.9	4.4
HPV-58	28	8.8	20.3	25.2	1.6	5.7	12.0
HPV-59	6	1.9	4.3	5.4	0.7	0.6	3.5
*Probable high-risk HPV genotype*							
HPV-66	25	7.9	18.1	54.3	1.5	5.3	11.1
HPV-68	26	8.2	18.8	56.5	1.5	5.7	11.4
*Low-risk HPV genotype*							
HPV-6/11	3	0.9	2.2	5.6	0.6	0.0	2.2
HPV-42	30	9.5	21.7	55.6	1.6	6.3	12.9
HPV-43	17	5.4	12.3	31.5	1.3	2.8	8.2
HPV-44	7	2.2	5.1	13.0	0.8	0.6	3.8
*Vaccine covered genotypes*							
HPV 16/18	26	8.2			1.6	5.0	11.7
HPV 6/11/16/18	29	9.1			1.7	6.0	12.6
HPV 6/11/16/18/31/33/45/52/58	90	28.4			2.5	23.3	33.2

*Because of the possibility of multiple infections, women may be counted more than once

^a^Percentages calculated using the total number of cases (N=317) as a common denominator

^b^Percentages calculated using the number of positive cases (N=138) as a common denominator

^c^Percentages calculated using the number of positive cases among the risk category as a common denominator

Among the LR HPV group the most common genotype was HPV-42 (9.5%, 95% CI 6.3–12.9) followed by HPV-43 (5.4%, 95% CI 2.8–0.8.2). HPV type 66 and HPV type 68 (probable high risk types), were detected 7.9% (95% CI 5.3–11.1) and 8.2% (95% CI 5.7–11.4), respectively. The more prevalent HR types were HPV 52 (18.3%, 95% CI 14.2–22.4), HPV 58 (8.8%, 95% CI 5.7–12.0), HPV 35 (5.4%, 95% CI 2.8–7.9), HPV 16 (4.4%, 2.2–6.6), HPV 18 (4.1%, 95% CI 2.2–6.3) and HPV 45 (4.1%, 95% CI 2.2–6.3). Among participants positive for HPV, 42.8% (n = 59) were infected with single HPV type infection and 57.2% (n = 79) were infected with multiple HPV types.

### HPV vaccine type prevalence

The prevalence of women with the bivalent vaccine type HPV (16/18) was 8.2% (95% CI 4.7–11.4%), quadrivalent vaccine-type HPV (6/11/16/18) was 9.1% (95% CI 5.7–12.3%) and nonavalent vaccine-type HPV (6/11/16/18/31/33/45/52/58) was 28.4% (95% CI 23.6–32.8%) (Table 3). The overall frequency of infections caused by HR HPV genotypes, covered by three vaccines (bivalent, quadrivalent and nonavalent) was lower (28.4%) than the overall frequency of all other HPVs detected in the study (45%), as some of the women had multiple infection with different HPV genotypes. Prevalence of HPV-16 and HPV-18 was 4.4% (14/317) and 4.1% (13/317), respectively. Prevalence of HPV 6/11 was 0.9% (3/317). Prevalence of additional five HPV genotypes included in nonavalent vaccine, HPV-31, 33, 45, 52 and 58 were 3.8% (12/317), 0.6% (2/317), 4.1% (13/317), 18.3% (58/317) and 8.8% (28/317) respectively. Table 3 shows, that among HR HPV group with single infection HPV-52 was more prevalent (n = 9), whereas among LR HPV group HPV-42 was more prevalent (n = 14).

**Table 3:** HPV genotype frequency and prevalence among single and multiple HPV infections observed in 138 positive women

S/N	HPV category	HPV genotype	Type of HPV infection						Total multiple
			Single infection	Multiple infection					
			1	2	3	4	5	6	
1	HR	16	3	4	1	4	1	1	11
2		18	8	2	0	1	2	0	5
3		31	2	1	2	2	4	1	10
4		33	0	2	1	0	0	0	2
5		35	6	6	2	2	1	0	11
6		39	1	2	1	3	3	0	9
7		45	1	2	1	3	3	3	12
8		51	0	2	0	3	2	0	7
9		52	10	21	19	5	3	0	48
10		56	3	2	2	1	0	0	5
11		58	2	8	6	6	4	2	26
12		59	0	2	2	1	0	1	6
1	PHR	66	1	3	4	8	6	3	24
2		68	5	6	6	5	3	1	21
1	LR	6/11	0	1	0	1	0	1	3
2		42	14	4	5	3	3	1	16
3		43	1	11	4	0	1	0	16
4		44	3	1	0	1	2	0	4

### Prevalence of HPV genotypes according to cervical cytology

Among participants with normal cytology results, 43.0% (129/300) were found to be positive for HPV infection (Table 4). Among women whose results were reported ASCUS/LSIL and HSIL/SCC, 66.7% (4/6) and 33.3% (1/3) were HPV positive, respectively. Majority of participants with normal cytology had multiple HPV infection (n = 73). Among cases with abnormal cytology report, only one had single HPV infection but the majority of abnormal cytology were infected with multiple genotypes (Table 4). Among women with normal cytology results, the most prevalent HR HPV type was HPV-52 (n = 55), followed by HPV-58 (n = 27). However, the most common LR was HPV-42 (n = 30). Among abnormal cytology results the most prevalent HR HPV type was also HPV-52 (n = 3), followed by HPV-45 (n = 2) and HPV-35 (n = 2). Nonavalent vaccine HPV genotypes were found among 50% (3/6) cases with ASCUS/LSIL and 33.3% (1/3) cases with HSIL/SCC.

**Table 4:** Prevalence of HPV infection and HPV genotype distribution in cytology outcome among participants

Parameter	Cytology results			
	Normal, n	ASCUS/LSIL, n	HSIL/SCC, n	Unsatisfactory, n
*Type of infection*				
Single	56	1	0	2
Multiple	73	3	1	2
*HPV genotype*				
**High-risk**				
HPV 16	10	0	1	3
HPV 18	13	0	0	0
HPV 31	10	1	0	1
HPV 33	0	1	0	1
HPV 35	15	2	0	0
HPV 39	9	0	1	0
HPV 45	11	2	0	0
HPV 51	7	0	0	0
HPV 52	55	3	1	0
HPV 56	7	0	0	1
HPV 58	27	0	1	0
HPV 59	6	0	0	0
**Probable high-risk**				
HPV 66	24	1	0	0
HPV 68	25	0	0	1
**Low-risk**				
HPV 6/11	3	0	0	0
HPV 42	30	0	0	0
HPV 43	17	0	0	0
HPV 44	7	0	0	0

### Associated risk factors

The results were further analyzed for cross-sectional associations between infection with HPV and sociodemographic, obstetric and behavioral characteristics, as well as the development of squamous intraepithelial lesions (Tables 5 and 6). Potentially significant associations were observed between infection with HPV and educational background (*p* < 0.001), age of first sexual intercourse (*p* = 0.016) and age of first pregnancy (*p* = 0.028).

**Table 5:** Crude odds ratio on the association between socio-demographic and obstetric characteristics and HPV positivity

Parameter	n (%)	Χ^2^ (*p* value)	Β (*p* value)	OR	95% CI
**Demographic characteristics**					
*Age group, years*		4.283 (0.232)			
**≤ **25	8 (2.5)			1	
25–44	215 (67.8)		− 1.332 (0.108)	0.264	0.052–1.337
45–64	87 (27.4)		− 1.495 (0.077)	0.224	0.043–1.176
≥ 65	7 (2.2)		− 2.015 (0.085)	0.133	0.013–1.318
*Education*		18.878 (**0.000**)			
< SHS	188 (59.3)			1	
≥ SHS	129 (40.7)		1.014 (**0.000**)	2.756	1.735–4.377
*Marital status*		0.003 (0.954)			
Married/cohabiting	205 (64.7)		− 0.014 (0.954)	0.986	0.620–1.569
Single/widowed/divorced	112 (35.3)			1	
*Occupation*		3.117 (0.077)			
Economically active^a^	275 (86.8)		0.620 (0.081)	1.859	0.927–3.728
Not economically active^b^	42 (13.2)			1	
**Obstetric characteristics**					
*Age at first pregnancy (years)*	12.508 (**0.028**)				
≤ 17	27 (8.5)			1	
18–21	97 (30.6)		1.605 (**0.006**)	4.976	1.601–15.470
22–25	76 (24.0)		1.377 (**0.020**)	3.961	1.247–12.587
> 25	59 (18.6)		1.579 (**0.009**)	4.852	1.492–15.771
Never pregnant	37 (11.7)		2.021 (**0.001**)	7.547	2.173–26.214
Do not remember	21 (6.6)		1.654 (**0.017**)	5.227	1.336–20.450
*Gravidae*		4.114 (0.128)			
0	37 (11.7)			1	
1	36 (11.4)		− 0.272 (0.563)	0.762	0.303–1.915
≥ 2	244 (77.0)		− 0.654 (0.067)	0.520	0.259–1.046
*Parity*		3.653 (0.301)			
0	63 (19.9)			1	
1	52 (16.4)		− 0.438 (0.249)	0.645	0.306–1.360
2–4	162 (51.1)		− 0.142 (0.634)	0.868	0.485–1.555
> 5	40 (12.6)		− 0.699 (0.097)	0.497	0.218–1.135

Bold font: *p* value <0.05

^a^Economically active—engages in paid work

^b^Not economically active—has no paid work, pensioner or housewife

**Table 6:** Crude odds ratio on the association between behavioral characteristics and HPV positivity

Parameter	n (%)	Χ^2^ (*p* value)	Β (*p* value)	OR	95% CI
*Age of coitarche (years)*	12.130 (**0.016**)				
≤ 15	22 (6.9)			1	
16–20	171 (53.9)		1.503 (**0.019**)	4.497	1.282–15.775
21–25	42 (13.2)		1.751 (**0.012**)	5.758	1.478–22.431
≥ 26	20 (6.3)		1.846 (**0.016**)	6.333	1.413–28.393
Do not remember	62 (19.6)		2.040 (**0.002**)	7.690	2.062–28.665
*Number of life time sex partners*	0.032 (0.858)				
1	112 (35.3)			1	
2+	205 (64.7)		0.043 (0.858)	1.043	0.656–1.661
*Tobacco use*		0.069 (0.793)			
Yes	4 (1.3)		0.184 (0.857)	1.201	0.162–8.895
No	313 (98.7)			1	
*Alcohol consumption*	2.037 (0.153)				
Yes	135 (42.6)		0.280 (0.236)	1.323	0.833–2.101
No	182 (57.4)			1	
*Condom use*		1.419 (0.234)			
Yes	108 (34.1)			1	
No	209 (65.9)		− 2.333 (0.340)	0.792	0.490–1.279

Bold font: *p* value <0.05

## Discussion

Epidemiological data on circulating type-specific HPV prevalence in a population is a very important rationale for introducing HPV vaccines and can provide an early measure of vaccine impact. In particular, it is important in countries where organized screening programs for the prevention of cervical cancer have not yet been established at scale. We conducted a multicenter descriptive study of the prevalence of HPV infection, and the vaccine-related genotype distribution among women presenting to reproductive health clinics. The prevalence of HPV infection detected in this study was 43.5% (95% CI 37.9–48.6). A similarly high prevalence of HPV in women in Sub-Saharan Africa has been reported by a number of studies [38–41]. A previous study done at the antenatal clinic in Korle-Bu Teaching Hospital among 93 healthy pregnant women reported HPV infection prevalence of 64.5% (n = 60) [42]. Yar and colleagues reported an overall HPV positivity for HIV-positive women and a control group as 86.9% and 56.0% respectively (for both case and control overall HPV prevalence was 72.0%) [40]. In general, differences in HPV prevalence reports may also be explained by the differences in study methodology: more specifically, the case volumes, the type of the case groups, study population and the method employed for HPV detection [43]. The present study relies on a highly-sensitive nested multiplex PCR (NMPCR) assay that combines degenerate E6/E7 consensus primers and type-specific primers for 16 HPV genotypes. This assay has been tried and proven to give consistent and reliable results when followed [37] and has been used in a couple of studies in Sub-Saharan Africa [34, 42, 44]. Polymerase chain reaction-based assays showed HPV prevalence of 40% in rural Mozambique [41], 31% in Harare, Zimbabwe [45], and 44% in Nairobi, Kenya. These figures suggest similar endemicity of HPV infection in the region.

Higher HPV prevalence may also be attributed to the nature of the study population and age range. Women of reproductive age may be more sexually active than women of other age-groups such as adolescent girls and elderly women. Available epidemiological data on HPV in high-risk population groups promote the idea that over-representation of high-risk women in general population studies may raise the observed prevalence. For instance, among HIV-positive and HIV-negative women recruited at the Cape-Coast Teaching Hospital, prevalence of HPV infection was reported as 75% and 42.6%, respectively [46]. In addition, HIV-positive women have higher rates of persistent HPV infections [40, 46].

Infection with more than a single genotype is a common feature of HPV infections [3, 47]. Cervical coinfection with multiple HPV types was observed for both HR HPV and LR HPV infections, and was supported by previous studies in Ghana [40, 42, 48] and sub-Saharan Africa [39, 49, 50]. Although co-infection of HPV genotypes occurs very frequently, the evidence suggests that the presence of multiple types does not especially influence clearance of HPV infections either by natural mechanism or vaccine-induced immunity [51, 52]. Rather, multiple infections occur as independent events sharing common transmission routes and having a similar profile of risk factors [53].

The more common HPV genotypes, from HR-HPV and PHR-HPV groups, identified among the study participants were HPV-52, HPV-58, HPV-66, HPV-68 and 35. In one Ghanaian study, Yar et al. [40] reported five predominant HR-HPV genotypes 58, 35, 68, 31 and 18, whereas Brandful et al. [42] reported 68, 66, 58, 35 and 56. Awua et al. [44], reported HP-16 (5.9%), HPV-35 (4.7%), HPV-40 (4.7%), HPV-45 (4.3%), HPV-58 (4.0%), HPV-18 (3.6%) to be the most prevalent HPV genotypes detected in self-collected specimens. Krings et al*.* [54] at North Tongu District of Ghana reported top five HPV genotypes: 16 (7.4%), 52 (7.2%), 35 (4.8%, 59 (4.7%) and 56 (3.9%). Concordance between studies on the distributions of observed genotypes strengthen our results and the conclusions that emanate from this work.

Additionally, the prevalence of HPV-16 and HPV-18 was 4.4% and 4.1% respectively. The higher prevalence of HPV-18 over HPV-16 in this study is similar to previous reports among available local studies in normal cervixes [40, 42, 55] and cancerous tissue [44, 56]. Although the proportions of specific genotypes differ, similar reports confirm that the most common genotypes detected in this study are equally prominent across other African countries both in women with normal cytology and in those with HSIL or worse [38, 41, 45, 49, 57]. It is possible that local differences in HPV distributions actually exist and must be continuously investigated in order to better understand the complex interplay of factors that shape HPV distributions in populations. However, it is also plausible that type-specific HPV prevalence may be influenced by the type of assay used and by the preponderance of multiple HPV infection in certain populations [45].

An important question which population HPV studies seek to answer pertains to the frequency of detection of HPV-16 and-18, the two most common high-risk genotypes prevented by available vaccines. The combined prevalence of HPV-16 and 18 in this study is similar to estimates from other local studies from Kumasi (6.2%) [32] and Accra (6.6%) [42]. The result of this study is consistent with other studies, which reported lower prevalence of these two vaccine-preventable genotypes in the general population compared to studies involving histologically confirmed cancer tissue [58–60]. These findings support the knowledge that HPV16 and HPV18 are mostly under-represented in women with normal cytology by comparison with their importance in severe cervical lesions and underscore their epidemiological reputation as more aggressive carcinogenic agents and justify the use of preventive vaccination in cancer prevention [5, 61, 62].

Since 2015, the Advisory Committee on Immunization Practices (ACIP) recommended an FDA approved nonavalent (9-valent) human papillomavirus (HPV) vaccine (9vHPV) (Gardasil 9, Merck and Co., Inc.) containing HPV-31, 33, 45, 52, and 58 VLPs in addition to the quadrivalent vaccine coverage [17, 18]. This new vaccine is hoped to expand the range of existing vaccines and protect even more women from HPV infections. Gardasil 9 vaccine includes five more HPV genotypes (HPV-52, 58, 45, and 31) covering the most common HR-HPV types found in our study among Ghanaian women. The high prevalence of nonavalent vaccine-preventable types coincidental with abnormal cytological findings taken together with previous reports of the distribution of vaccine preventable HPV genotypes in malignant cervical tissue [56, 58] show that expanded genotype vaccines may be more beneficial than previous vaccines in this population.

Earlier age at sexual debut puts women at greater risk for HPV infection [63]. An association between young age and cervical HPV infections is generally attributed to a higher susceptibility to the infection at the beginning of sexual activity, with peak prevalence in younger women and progressive decline with increasing age [64].

In this study we found that although there was a high burden of HPV infection in young adult women (18–25 years) (Additional file 1), the overall prevalence of infection from vaccine-type HPVs in sexually active women was low in the age group less than 25 years (0% for HPV-16/18, 0% for HPV-6/11/16/18 and 1.3% (4/318) for HPV-6/11/16/18/31/33/45/53/58) and a wide margin of interventions with vaccine primary prophylaxis beyond the preadolescent girls aged 9–12 years, who are actually the target for vaccine, could be expected.

In this work, an important socio-demographical risk factor associated with of HPV DNA infection was education. In general, low educational status is thought to indicate poor knowledge and compliance for safer sexual behavior; including number of sex partners and use of protective condoms [65]. Our results suggest that higher education may not necessarily be indicative of knowledge of HPV or safer sexual behavior. Our data showed that women who were active (have any job, government or private) had 2 times higher risk to be infected with HPV, than those who were not active (do not have any job). But our study did not look at the association of prevalence of HPV infection with socio-economic status of the women. Though this contradiction would be hard to explain, it may be related to difference in a risk behavior correlated with education.

## Limitations

The study used a convenient hospital-based sampling. The benefits of this approach include time and cost-savings. However, there are many setbacks with this approach to sampling. These include a high chance of bias and the applicability of findings to other population groups such as community-dwelling women or the general population of women. Bias was minimized by excluding previously screened women and skipping every third woman presenting to the clinic. These factors should always be borne in mind when interpreting study results.

## Conclusion

Vaccine preventable high-risk HPV types represent 8.2% (HPV-16 and 18) and 9.1% (HPV-6, 11, 16 and 18) of the burden of HPV infections in the population studied. Although the impact of universal vaccination with existing vaccines would be much greater with data from women with pre-cancerous lesions, our data shows that the nonavalent vaccine targeted at HR HPV-16, -18, -31, -33, -45, -52 and -58, would cover 28.4 0% of HPV infections. As the country prepares to achieve 90% universal vaccine coverage, the role of expanded-scope vaccines that confer immunity against region-specific oncogenic HPV types can be essential. It is an urgent need to introduce the nonavalent vaccine to the country at scale.

## Supplementary Information

**Additional file 1**. Prevalence of vaccine-preventable human papillomaviruses stratified according to demographic and reproductive characteristics.

## Data Availability

The dataset supporting the conclusions of this article is included within the article and its additional files. More detailed data can be obtained from the Committee on Human Research Publication and Ethics (CHRPE), Kwame Nkrumah University of Science and Technology, School of Medical Sciences (KNUST-SMS) and Komfo Anokye Teaching Hospital (KATH) board.

## Abbreviations

ASCUS: Atypical squamous cells of undetermined significance
GAVI: Global alliance for vaccines initiative
HPV: Human papillomavirus
HR-HPV: High risk human papillomavirus
HSIL: High grade squamous intraepithelial lesion
ICC: Invasive cervical cancer
LR-HPV: Low risk human papillomavirus
LSIL: Low-grade squamous intraepithelial lesion
PHR-HPV: Probable high risk human papillomavirus
Pap: Papanicolaou
SPSS: Statistical package for the social scientists
SCC: Squamous cervical cancer
VIA: Visual inspection with acetic acid
WHO: World health organization
