# Supplementary material for: Prevalence of vaccine and non-vaccine human papillomavirus types among women in Accra and Kumasi, Ghana: a cross-sectional study
Source: BMC Womens Health. 2021 Oct 26;21:372. doi: 10.1186/s12905-021-01511-1 (PMC8549380; doi:10.1186/s12905-021-01511-1)
Supplement: Supplementary file 1 — Additional file 1. Prevalence of vaccine-preventable human papillomaviruses stratified according to demographic and reproductive characteristics. [file 12905_2021_1511_MOESM1_ESM.docx]

**Prevalence of vaccine and non-vaccine human papillomavirus types among women in Accra and Kumasi, Ghana: a cross-sectional study**

Oksana Debrah, Francis Agyemang-Yeboah, Emmanuel Timmy Donkoh, Richard Harry Asmah

**S1 Table: Demographic characteristics & Prevalence of human papilloma virus (HPV) and vaccine covered genotypes among women**

| **Characteristics** | **Number** | **HPV positive, any type**  **N (%)^1^** | **HPV 16/18**  **N (%)^2^** | **HPV 6/11/16/18**  **N (%)^2^** | **HPV 6/11/16/18/31/33/45/52/58**  **N (%)^2^** |
| --- | --- | --- | --- | --- | --- |
| **Age group, years** |  |  |  |  |  |
| **≤** 25 | 8 | 6 (75.0) | 0 (0) | 0 (0) | 4 (50.0) |
| 25 - 44 | 215 | 50 (46.3) | 18 (8.4) (16.7) | 19 (8.8) | 65 (30.2) |
| 45 - 64 | 87 | 45 (42.1) | 8 (9.2) | 9 (10.3) | 19 (21.8) |
| ≥ 65 | 7 | 2 (28.6) | 0 (0) | 1 (14.3) | 2 (28.6) |
| **Education** |  |  |  |  |  |
| < SHS | 188 | 63 (33.5) | 8 (4.3) | 10 (5.3) | 35 (18.6) |
| ≥ SHS | 129 | 75 (58.1) | 18 (14.0) | 19 (14.7) | 55 (42.6) |
| **Marital status** |  |  |  |  |  |
| Married /Cohabiting | 205 | 49 (43.8) | 15 (7.3) | 16 (7.8) | 55 (26.8) |
| Single/Widowed/Divorced | 112 | 89 (43.5) | 11 (9.8) | 13 (11.6) | 35 (31.3) |
| **Occupation** |  |  |  |  |  |
| ^1^Economically active | 275 | 125 (45.5) | 26 (9.5) | 28 (10.2) | 81 (29.5) |
| ^2^Not economically active | 42 | 13 (31.0) | 0 (0) | 1 (2.4) | 9 (21.4) |
| *****Row percentages calculated with reference to total number of case in a row;  ^1^Economically active - have any job, ^2^Not economically active – have no job, pensioner or housewife | | | | | |

**S2 Table: Obstetric characteristics & Prevalence of human papilloma virus (HPV) and vaccine covered genotypes among women**

| **Characteristics** | **Number** | **HPV positive**  **total**  **N (%)^1^** | **HPV 16/18**  **N (%)^2^** | **HPV 6/11/16/18**  **N (%)^2^** | **HPV 6/11/16/18/31/33/45/52/58**  **N (%)^2^** |
| --- | --- | --- | --- | --- | --- |
| ***Age at first pregnancy (years)*** | | | | |  |
| ≤ 17 | 27 | 4 (14.8) | 0 (0) | 0 (0) | 3 (11.1) |
| 18 - 21 | 97 | 45 (46.4) | 7 (7.2) | 9 (9.3) | 27 (27.8) |
| 22 - 25 | 76 | 31 (40.8) | 7 (9.2) | 7 (9.2) | 19 (25.0) |
| > 25 | 59 | 27 (45.8) | 7 (11.9) | 7 (11.9) | 20 (33.9) |
| Never pregnant | 37 | 21 (56.8) | 4 (10.8) | 5 (13.5) | 16 (43.5) |
| Do not remember | 21 | 10 (47.6) | 1 (4.8) | 1 (4.8) | 5 (23.8) |
| ***Gravidae*** |  |  |  |  |  |
| 0 | 37 | 21 (56.8) | 4 (10.8) | 5 (13.5) | 16 (43.2) |
| 1 | 36 | 18 (50.0) | 5 (13.9) | 5 (13.9) | 13 (38.1) |
| ≥ 2 | 244 | 99 (40.6) | 17 (7.0) | 19 (7.8) | 61 (25.0) |
| ***Parity*** |  |  |  |  |  |
| 0 | 63 | 31 (49.2) | 8 (12.7) | 9 (4.3) | 25 (39.7) |
| 1 | 52 | 20 (38.5) | 3 (5.8) | 3 (5.8) | 10 (19.2) |
| 2-4 | 162 | 74 (45.7) | 14 (8.6) | 15 (9.3) | 49 (30.2) |
| >5 | 40 | 13 (32.5) | 1 (2.5) | 2 (5.0) | 6 (15.0) |
| ***Cytological Result*** |  |  |  |  |  |
| Normal | 300 | 129 (43.0) | 22 (7.3) | 25 (8.3) | 83 (27.7) |
| ASCUS/ LSIL | 6 | 4 (66.7) | 0 (0) | 0 (0) | 3 (50.0) |
| HSIL/ SCC | 3 | 1 (33.3) | 1 (33.3) | 1 (33.3) | 1 (33.3) |
| Unsatisfactory | 8 | 4 (50.0) | 3 (37.5) | 3 (37.5) | 3 (37.5) |
| *****Row percentages calculated with reference to total number of cases in a row | | | | | |

**S3 Table: Behavioral characteristics & Prevalence of human papilloma virus (HPV) and vaccine covered genotypes among women**

| **Characteristics** | | **Number** | **HPV positive total**  **N (%)^1^** | | **HPV 16/18**  **N (%)^2^** | | **HPV 6/11/16/18**  **N (%)^2^** | **HPV 6/11/16/18/31/33/45/52/58**  **N (%)^2^** |
| --- | --- | --- | --- | --- | --- | --- | --- | --- |
| **Age of coitarche (years)** | | | | | | | |  |
| ≤ 15 | | 22 | 3 (13.6) | | 0 (0) | | 0 (0) | 2 (9.1) |
| 16 - 20 | | 171 | 71 (41.5) | | 14 (8.2) | | 17 (9.9) | 47 (27.5) |
| 21 - 25 | | 42 | 20 (47.6) | | 5 (11.9) | | 5 (11.9) | 15 (35.7) |
| ≥ 26 | | 20 | 10 (50.0) | | 3 (15.0) | | 3 (15.0) | 7 (35.0) |
| Do not remember | | 62 | 35 (54.8) | | 4 (65.0) | | 4 (65.0) | 62 (100) |
| **Number of life time sex partners** | | | | | | | |  |
| 1 | | 112 | 48 (42.9) | | 7 (6.3) | | 8 (7.1) | 27 (24.1) |
| 2+ | | 205 | 90 (43.9) | | 19 (9.3) | | 21 (10.2) | 63 (30.7) |
| **Tobacco use** | | | | | | | | |
| Yes | 4 | | | 2 (50.0) | 0 (0.0) | 0 (0.0) | | 1 (25.0) |
| No | 313 | | | 136 (43.5) | 26 (8.3) | 29 (9.3) | | 69 (28.4) |
| **Alcohol consumption** | | | | | | | | |
| Yes | 135 | | | 65 (48.1) | 12 (8.9) | 13 (9.6) | | 44 (32.6) |
| No | 182 | | | 73 (40.1) | 14 (7.7) | 16 (8.8) | | 46 (25.3) |
| **Condom use** |  | | |  |  |  | |  |
| Yes | 108 | | | 52 (48.1) | 12 (11.1) | 13 (12.0) | | 36 (33.3) |
| No | 209 | | | 86 (41.1) | 14 (6.7) | 16 (7.7) | | 54 (25.8) |
| *Row percentages calculated with reference to total number of case in a row | | | | | | | | |
